# Metabolic shift in density-dependent stem cell differentiation

**DOI:** 10.1186/s12964-017-0173-2

**Published:** 2017-10-20

**Authors:** Simar J. Singh, William Turner, Drew E. Glaser, Kara E. McCloskey, Fabian V. Filipp

**Affiliations:** 1 Systems Biology and Cancer Metabolism, Program for Quantitative Systems Biology, University of California Merced, 2500 North Lake Road, Merced, CA 95343 USA; 2 Program for Biological Engineering and Small-scale Technologies, School of Engineering, University of California Merced, 5200 North Lake Road, Merced, CA 95343 USA

**Keywords:** Stem cells, Differentiation, Vascular fate, Cell seeding density, Systems biology, Metabolism, NMR, Metabolomics, Fluorescence-activated cell sorting, Flow cytometry, Cell adhesion, Cell contact, Cell communication, Microenvironment, Cancer stem cells, Embryonic stem cells, Vascular progenitor cells, Endothelial cells

## Abstract

**Background:**

Vascular progenitor cells (VPCs) derived from embryonic stem cells (ESCs) are a valuable source for cell- and tissue-based therapeutic strategies. During the optimization of endothelial cell (EC) inductions from mouse ESCs using our staged and chemically-defined induction methods, we found that cell seeding density but not VEGF treatment between 10 ng/mL and 40 ng/mL was a significant variable directing ESCs into FLK1^+^ VPCs during stage 1 induction. Here, we examine potential contributions from cell-to-cell signaling or cellular metabolism in the production of VPCs from ESCs seeded at different cell densities.

**Methods:**

Using 1D ^1^H-NMR spectroscopy, transcriptomic arrays, and flow cytometry, we observed that the density-dependent differentiation of ESCs into FLK1^+^ VPCs positively correlated with a shift in metabolism and cellular growth.

**Results:**

Specifically, cell differentiation correlated with an earlier plateauing of exhaustive glycolysis, decreased lactate production, lower metabolite consumption, decreased cellular proliferation and an increase in cell size. In contrast, cells seeded at a lower density of 1,000 cells/cm^2^ exhibited increased rates of glycolysis, lactate secretion, metabolite utilization, and proliferation over the same induction period. Gene expression analysis indicated that high cell seeding density correlated with up-regulation of several genes including cell adhesion molecules of the notch family (NOTCH1 and NOTCH4) and cadherin family (CDH5) related to vascular development.

**Conclusions:**

These results confirm that a distinct metabolic phenotype correlates with cell differentiation of VPCs.

## Background

Vascular progenitor cells (VPCs) and endothelial cells (ECs) are desirable cell sources for cellular therapeutic and tissue engineering strategies including: peripheral vascular disease [1, 2], severe ischemic heart disease [3, 4] and lining the lumens of small diameter vascular grafts in order to minimize thrombosis or arteriosclerosis [5, 6]. In cancer, the vascular niche promotes cancer stem cells (CSCs) and is enriched with CSC-derived ECs, which promote tumor invasion and metastasis [7]. VPCs are important for maintenance of the stemness of normal adult stem cells, including self-renewal, undifferentiated status, and dormancy. However, it is sometimes difficult to obtain sufficient numbers of proliferating VPCs and ECs, especially from aged adults and diseased patients [6]. Alternatively, embryonic stem cells (ESCs) and induced pluripotent stem cells (iPSCs) with their unlimited capacity for self-renewal, are considered excellent cell sources in a variety of cell-based therapies. In addition to their growing therapeutic applications, these cell sources in combination with derived VPCs and ECs can also serve as representative in vitro models of vascular development.

During early stages of vascular development, signaling from vascular endothelial growth factor (VEGF = VEGFA, vascular endothelial growth factor A, GeneBank: 7422) and the VEGF receptor, FLK1 (FLK1 = VEGFR = KDR, kinase insert domain receptor, GeneBank: 3791) promotes ventral mesoderm and hematopoietic fate [8–10] leading to activation of the mitogen activated protein kinase pathway [11]. Endothelial, hematopoietic, and smooth muscle cells have been derived from outgrowths of FLK1^+^ VPCs, making this VEGF receptor a hallmark for identification of VPCs [12]. However, despite our growing understanding of the critical biochemical factors in development, the precise timing and quantitative levels of EC induction/activation for directing vascular fate from ESCs in vitro have remained confounding. This is complicated by the inherent variability in kinetic and autocrine signaling from ESC line-to-ESC line [13]. For example, the optimal time to induce the mouse D3-ESC line into FLK1^+^ VPCs has been reported to occur at day 4 (FLK1^+^ = FLK1 positive = VEGFR expressing cells) [14–16], while the optimal time for the corresponding mouse R1-ESC line has been reported at day 2 [17]. Additionally, while VEGF is the most published growth factor associated with directing EC differentiation, published treatment levels vary between 20 ng/mL and 50 ng/mL [12, 15, 18]. Matrix signaling is also an important signal in stem cell fate, but studies on this topic have also been conflicting. For example, it has been reported that collagen type-IV directs a higher percentage of ECs [12, 15, 18]. However, more recent studies show fibronectin promotes increased cell adhesion and/or proliferation, generating greater numbers of VPCs and ECs compared with collagen-type IV [1, 17]. Moreover, increasing evidence supports a role for modified cellular metabolism in the regulation of stem cell self-renewal, specification, and plasticity in cancer and development [19–21]. Despite this growing understanding of cellular metabolism as a regulator of cell function, the role of cell seeding density in metabolic alterations supporting vascular fate is not defined.

**Fig. 1:**
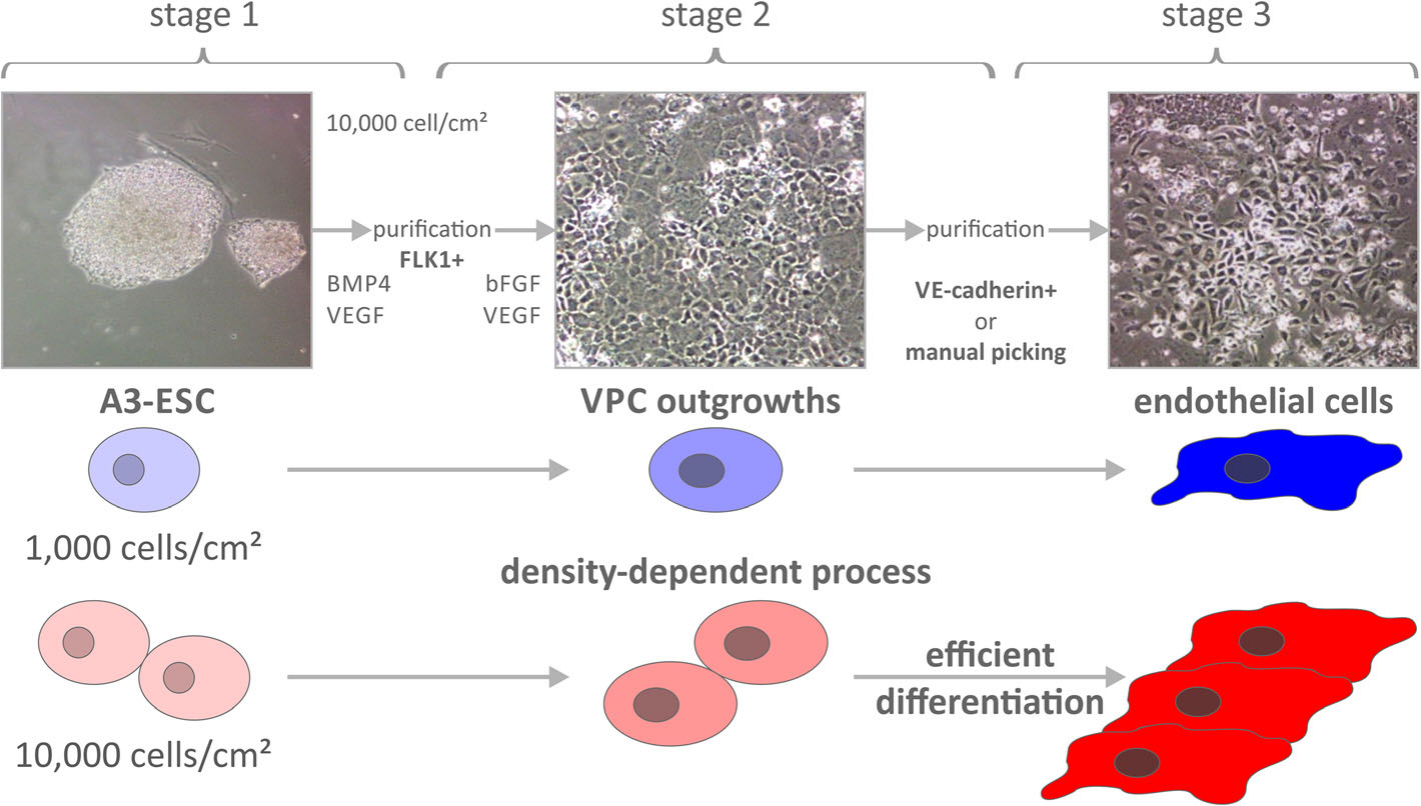
Higher seeding density yields higher expression of differentiation marker FLK1 (FLK1 = VEGFR = KDR, VEGF receptor, kinase insert domain receptor). Schematic of staged differentiation of embryonic stem cells (ESCs) into vascular progenitor cells (VPCs) and endothelial cells (ECs) with representative micrographs from A3-ESCs seeded at 10,000 cells/cm^2^. Stage 1 involves the culturing of ESCs on fibronectin in the presence of BMP4 (bone morphogenetic protein 4) and VEGF (vascular endothelial growth factor). At the completion of Stage 1, resulting VPCs are assayed for FLK1 receptor expression by an anti-FLK1 antibody

Therefore, using our established staged differentiation methodology and chemically-defined media formulations (Fig. 1a), we examined a number of combinatorial variables (induction time, VEGF treatment, matrix signaling, and cell seeding density) for directing the generation of VPCs (stage 1). The results indicated that cell seeding density was a significant factor in the first stages of induction of ESCs into VPCs, especially in the A3-ESC cell line [22] generated by our own laboratory. Therefore, we set out to further examine the underlying mechanisms related to density-dependent differentiation in this ESC line.

## Methods

### Embryonic stem cell culture

Mouse A3-ESCs were extracted, generated, and cultured at 3,000/cm^2^ on inactivated mouse embryonic fibroblasts (MEFs; 20,000/cm^2^) [22]. Prior to induction, the A3-ESCs are purified from MEFs by gravity separation followed by MEF adhesion to tissue culture dishes for 1–2 hours and passaged onto 0.5% gelatin-coated plates in ESC culture media containing: Knockout Dulbecco’s Modified Eagle Medium (KO-DMEM; Invitrogen), 15% Knockout Serum Replacer (KSR; Invitrogen), 1Χ penicillin-streptomycin (Invitrogen), 1Χ non-essential amino acids (Invitrogen), 2 mM L-glutamine (Invitrogen), 0.1 mM 2-mercaptoethanol (Calbiochem), 2000 units/mL of leukemia inhibitory factor (LIF-ESGRO; Chemicon), and 10 ng/mL of bone morphogenetic protein 4 (BMP4, GeneBank: 652) (R&D Systems). Full media changes occurred every other day and cells were passaged every 4–5 days.

### Induction of FLK1^+^ VPCs

A3-ESCs were harvested and plated at either 1,000, 5,000 or 10,000 cells/cm^2^ in 12-well cell culture dishes, coated with 50 ng/mL fibronectin (BD Biosciences), and fed our induction media: alpha-minimal essential medium (MEM; Corning), 20% KSR (Invitrogen), 1Χ penicillin-streptomycin (Invitrogen), 1Χ nonessential amino acids (Invitrogen), 2 mM L-glutamine (Invitrogen), 0.05 mM 2-mercaptoethanol (Calbiochem), and 5 ng/mL BMP4 (R&D Systems), and 0 to 30 ng/mL of VEGF (R&D Systems) without media change for 4 days. Experiments were conducted in triplicate (*N* = 3) allowing for analysis of variance. The assessment of stage 1 VPCs, which are not contact-inhibited, was quantified by the percentage of FLK1^+^ cells over time, previously shown to correlate with down-regulation of the pluripotent stem cell marker POU class 5 homeobox 1 (POU5F1 = OCT3/4, GeneBank: 5460) over the same time period [22].

### Characterization of VPCs

Adherent cells were harvested using Cell Dissociation Buffer (Invitrogen), fixed in 4% paraformaldehyde (Tousimis), rinsed 2Χ with phosphate buffered saline (PBS), blocked using 0.5% donkey serum (Fitzgerald) and 1% bovine-serum albumin (Sigma) for 1 h at room temperature, and stained with Alexa Fluor 647®-conjugated anti-FLK1 antibody (Biolegend) at 1:100 and allowed to incubate overnight at 4 °C. Cells were rinsed 2Χ with PBS before being analyzed on an LSR II flow cytometer (BD Biosciences) and FloJo Software (TreeStar) at 1, 2, and 3 days post induction of differentiation. Samples were analyzed in triplicate (*N* = 3) for each data point.

### Exometabolome analysis

Triplicate samples of conditioned induction media (*N* = 3) were harvested at 1, 2 and 3 days post-induction and stored at −80 °C. Prior to 1D ^1^H-NMR spectroscopy metabolomics analysis, supernatants were extracted using 1:1 cold methanol (BDH 67-56-1) and chloroform (Amresco 0757) mixture [7]. The extracts were cleared by centrifugation at 14,000 g and the aqueous phase was collected. Freeze-dried metabolite samples were resuspended in 200 μL of H20 with 5% D2O spiked with 0.75% 3-(trimethylsilyl)propanoic-2,2,3,3-d4 acid (TSP) (Sigma 293040) to a final concentration of 2.409 mM into 3 mm NMR tubes (Norrell C-S-3-HT-7). Spectra were recorded using 1D ^1^H excitation sculpting at 512 scans, d1 = 1 s, 1H pulse 11.0 μs, power level of shaped pulse 25.55db and an experimental time of 10 min at 300 K at an Avance II 600-MHz spectrometer fitted with a cryogenic probe operating with TOPSPIN 2.0 (Bruker BioSpin GmbH). All spectra were automatically phased, baseline corrected and referenced to TSP (δ 0.00 ppm) using Chenomx NMR spectroscopy suite 8.1 (Chenomx Inc). Metabolite concentrations were quantified on the basis of matching chemical shifts and multiplicities to the Chenomx reference compound library. Exometabolome analysis by NMR spectroscopy provides direct comparison of absolute metabolite concentrations of analytes. Not surprisingly, the amount of metabolites excreted or taken up scales with the initial seeding density. Therefore, by normalizing each time point to the first time point post-induction, dynamic information of the system can be obtained. The total cell count prior seeding was obtained in triplicate (*N* = 3) by analyzing cells accurately using multifocal plane analysis in the TC20 automated cell counter (Biorad).

### Cell size and proliferation

Cell diameter and proliferation rates were measured over the 3 days of VPC induction using an automated image-based cytometer. Cells were harvested using 0.25% trypsin-EDTA (Corning) from fibronectin coated cultures dishes at 1, 2 and 3 days post induction of differentiation, stained with trypan blue, and pipetted into disposable counting chambers for counting and image analysis. Cell diameter measurements of live differentiating ESCs were obtained in the TC20 automated cell counter (Biorad). Multi-planar bright-field digital images were automatically collected, quantified, and assessed for cell number and diameter. Cell proliferation rates were calculated and densities validated from the live cells per dish (*N* = 6) over the 3 days of VPC induction.

### Differential gene expression

Total RNA was extracted from undifferentiated ESCs as well as from cells 3 days post induction of differentiation using TRIzol (Sigma T9424). At least three biological replicates of RNA samples were analyzed per condition. The concentration of RNA was determined using a Nanodrop spectrophotometer (Thermo Scientific). Two micrograms of RNA was processed with the RT^2^ profiler array PAMM-146Z (Qiagen SABiosciences) and used to synthesize cDNA using the RT^2^ SYBR green master mix (Qiagen SABiosciences) in a 7300 real-time (RT) quantitative polymerase chain reaction (QPCR) System (Applied Biosystems). Gene expression profiles were analyzed using the ΔΔCT method. RT QPCR threshold cycle (CT) values were normalized using five different housekeeping genes (HKG), ACTB, actin beta, GeneBank: 60, B2M, beta-2-microglobulin, GeneBank: 567, GAPDH, glyceraldehyde-3-phosphate dehydrogenase, GeneBank: 2597, GUSB, glucuronidase beta GeneBank: 2990, and HSP90AB1, heat shock protein 90 kDa alpha family class B member 1, GeneBank: 3326. The difference threshold cycle value (ΔCT) of any gene of interest (GOI) to the average housekeeping value was calculated using the formula ΔCT(GOI) = CT(GOI) — AVERAGE(CT(HKG)) for ESCs, differentiating cells at seeding density of 1,000 cells/cm^2^ and 10,000 cells/cm^2^. In addition, change in gene expressions of any gene of interest was monitored by calculating ΔΔCT(GOI) = ΔCT(GOI-10 K) — ΔCT(GOI-1 K). RT^2^ gene array profiles were normalized, separated according to differential expression between the two seeding densities in univariate T-tests with a random variance model using a *p*-value cut-off below 0.05, and ranked with LOG2 fold-change of specimen seeded at 10,000 cells/cm^2^ in comparison to 1,000 cells/cm^2^ considered significant.

### Differential protein expression analysis

Induced VPCs originally seeded as ESCs at 1,000 cells/cm^2^ or 10,000 cells/cm^2^ were harvested 3 days post induction. Cells were fixed, washed, and blocked in PBS supplemented with 2% fetal bovine serum (FBS; Corning 35-010-CV). Cells were incubated light-protected at 4 °C for 1 h with the following antibodies and staining reagents: FLK1 PerCP (Biolegend 121915), CDH2 rabbit polyclonal (Abcam ab12221), CDH5 (CD144) brilliant violet 421 (Biolegend 138013), and Fixable Viability Dye eFluor780 (eBioscience 65-0865-14). After washing, cells were incubated light protected at 4 °C for 1 h with FITC conjugated Donkey Anti-Rabbit IgG pre-adsorbed (Abcam ab7079). Samples were rinsed twice with PBS supplemented with 2% FBS before being analyzed on an LSR II flow cytometer (BD Biosciences) at a flow rate at least 500 events per second. 100,000 events per sample were recorded and samples were analyzed in triplicate (*N* = 3) for each data point. FloJo Software (TreeStar) was used for data analysis. Dead cells were gated out from analysis based on Viability Dye eFlour780 reactivity. FLK1^+^ cells were then analyzed for FITC (CDH2) and Brilliant Violet 421 (CDH5) fluorescence and the percentage of Flk1^+^/CDH2^+^CDH5^+^ cells were compared between low density and high density groups.

## Results

### Characterization of differentiated FLK1^+^ VPCs

**Fig. 2:**
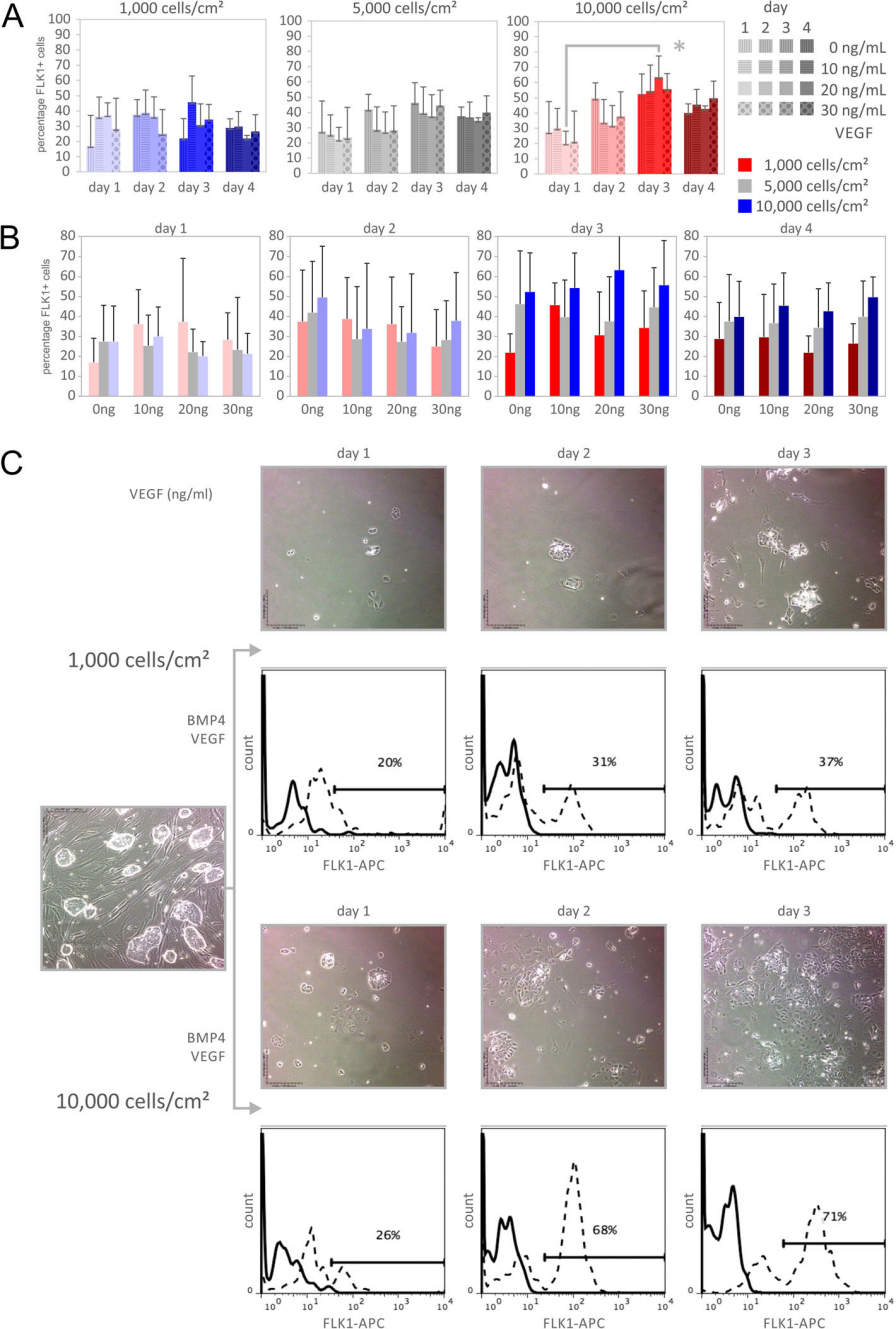
Higher seeding density yields higher expression of differentiation marker FLK1 (FLK1 = VEGFR = KDR, kinase insert domain receptor). The effect of (**a**) seeding density, (**b**) vascular endothelial growth factor (VEGF) concentration, and induction time on the percentage of FLK1^+^ vascular progenitor cells (VPCs) generated from embryonic stem cells (ESCs). An induction time of three days combined with a seeding density of 10,000 cells/cm^2^ resulted in high percentages of FLK1^+^ cells regardless of VEGF concentration. **c** Phase contrast images and flow cytometic cell scanning histograms of FLK1 expression of ESCs induced on 50 ng/mL fibronectin with 20 ng/mL VEGF treatment. *Upper panel* shows time course following seeding densities of 1,000 cells/cm^2^. *Lower panel* shows time course following seeding densities of 10,000 cells/cm^2^. By day 3 post induction, the majority of cells seeded at 10,000 cells/cm^2^ exhibit FLK1 receptor expression. In contrast, cells initially seeded at 1,000 cells/cm^2^ exhibit less FLK1 receptor expression and exhibit fewer cell clusters at day 3

Induction of mouse A3-ESCs [22] into VPCs was examined over a range of seeding densities, VEGF treatment levels, and time (Fig. 2a). The greatest number of FLK1^+^ cells was generated on day 3, with a reduction at day 4. Although the VEGF treatment levels led to variable results, the greatest number of FLK1^+^ VPCs was consistently and statistically significant in cultures seeded at the highest seeding density (Fig. 2a-b) while cells initially seeded at 1,000 cells/cm^2^ generated significantly fewer FLK1^+^ cells. Bright field microscopy revealed that after three days, the 10,000 cells/cm^2^ seeding density remained subconfluent (Fig. 2c).

### Metabolic shift during density-dependent differentiation

**Fig. 3:**
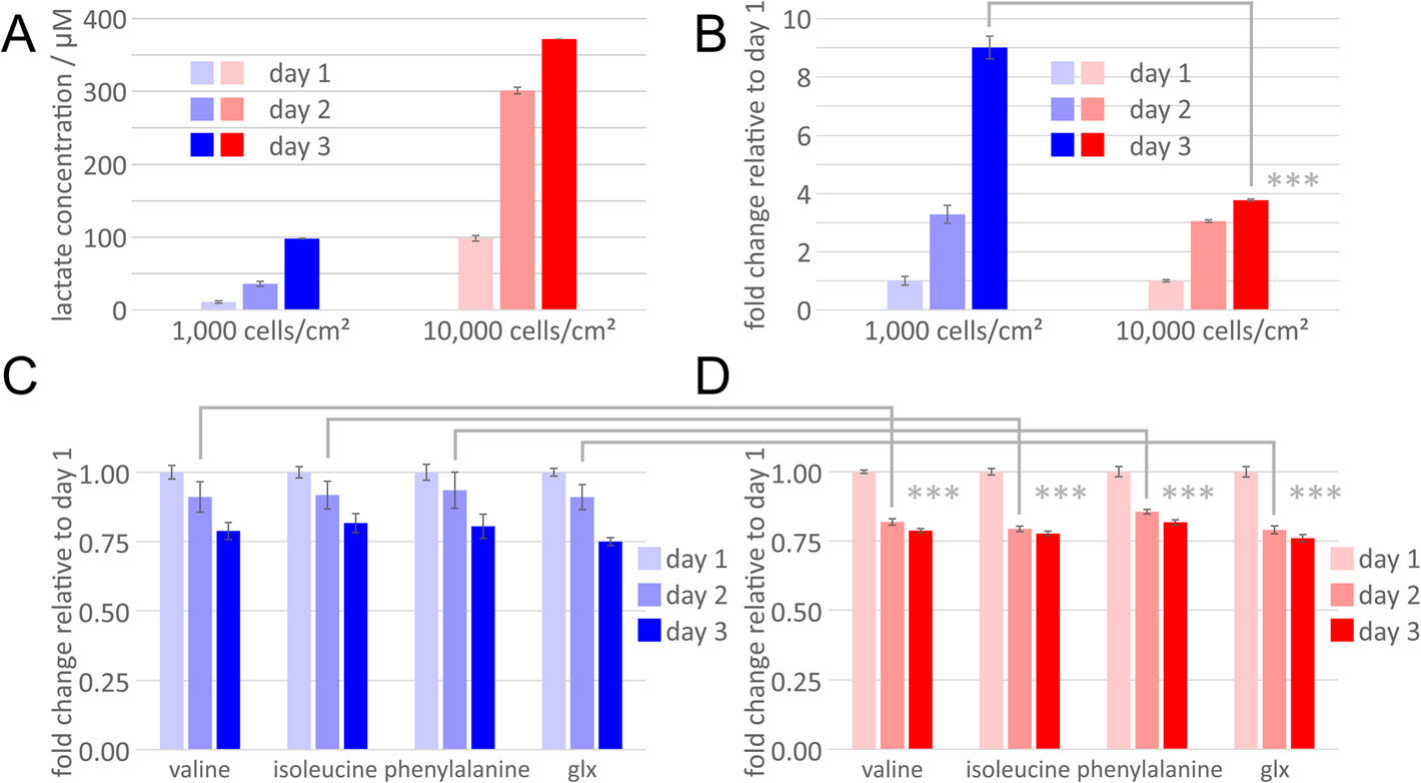
Density-dependent shift of metabolic rate. 1D ^1^H-NMR spectroscopic exometabolome analysis of conditioned media from induced embryonic stem cells (ESCs) initially seeded at 1,000 cells/cm^2^ (*blue*) and 10,000 cells/cm^2^ (*red*). **a** By day 3, cells seeded at higher density reduce production of lactate whereas cells initially seeded at low density continue to increase lactate production and exhibit a significantly higher fold increase in lactate abundance between days 1 and 3 (9.0 vs 3.8; *** *p*-value < 0.001). **b** Fold change of lactate production relative to day 1. **c** Amino acid uptake of valine, isoleucine, phenylalanine, and glutamine/glutamate (glx) significantly increases in the low density group after two days of induction (*** *p*-value < 0.001). **d** Amino acid uptake plateaus between 2 and 3 days post induction in the higher density group. Fold change of amino acid uptake relative to day 1

To identify density-dependent changes in cellular metabolism during differentiation, we measured metabolite abundance within conditioned media using 1D ^1^H-NMR spectroscopy. This exometabolome analysis provides insights into metabolite utilization and secretion. A reduction in metabolite abundance is consistent with cellular uptake from our chemically defined induction media, whereas an increase in abundance correlates with active production and extracellular secretion. Of the metabolites in the differentiation media profiled, only lactate exhibited an increase in abundance. Cells seeded at a density of 10,000 cells/cm^2^ displayed a rapid increase in lactate production between days 1 and 2, which then slowed between days 2 and 3 (Fig. 3a-b). Conversely, cells grown at a density of 1,000 cells/cm^2^ produce, on a per cell basis, comparatively more lactate, and exhibit a significant increase in lactate abundance between days 1 and 3 (9.0 vs 3.8; *p*-value < 0.001) (Fig. 3a-b). The same trend is seen in metabolite utilization. Cells grown at a density of 10,000 cells/cm^2^ exhibit higher rates of metabolite utilization between day 1 and day 2, and much lower utilization between days 2 and 3 (Fig. 3c-d). In contrast, cells seeded at lower density increase their metabolite uptake over time, exhibiting their highest levels of utilization between days 2 and 3 (Fig. 3c-d).

### Differentiation correlates with increased cell size and reduced proliferation

**Fig. 4:**
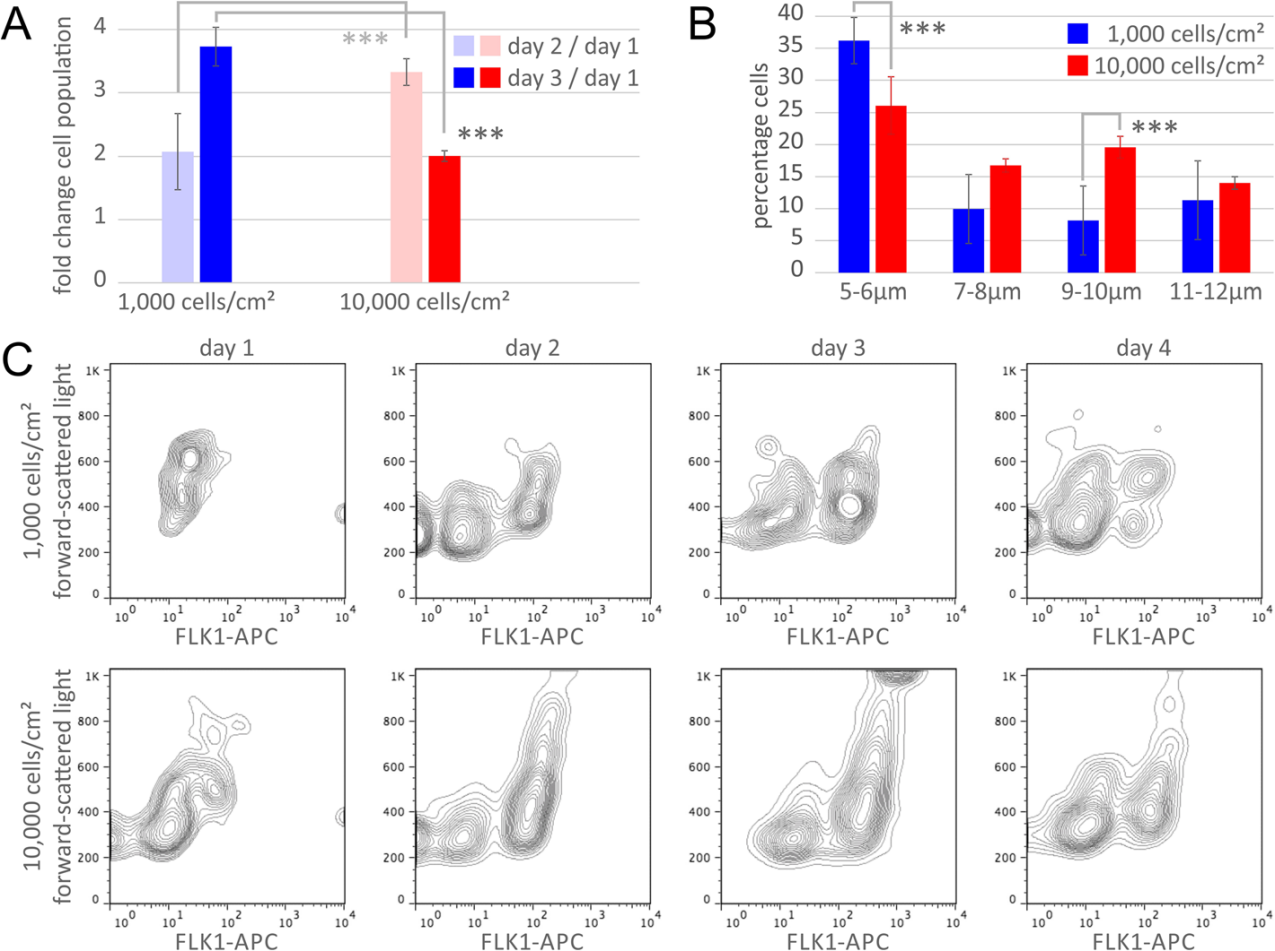
Density-dependent shift of proliferation and cell diameter. **a** Proliferation rate significantly slows at day 3 in cells seeded at 10,000 cells/cm2 (*red*) but increases in cells seeded at 1,000 cells/cm^2^ (*blue*; fold increase of 2.1 vs 3.7; *** *p*-value < 0.001). **b** Higher density cells have a greater percentage of cells with large diameter (9–10 μm, 19.6% vs 8.2%; *** *p*-value < 0.001) and fewer small diameter cells (5–6 μm, 26.1% vs 36.2%; *** *p*-value < 0.001). **c** Flow cytometric cell scanning contour plot indicating 10,000 cells/cm^2^ seeding density results in a greater proportion of cells exhibiting high forward scatter and FLK1 allophycocyanin conjugate (APC) positivity

To determine whether the observed shift in metabolite utilization coincides with a change in cellular proliferation, we measured the number of live cells present for both seeding densities following induction of differentiation. Cells induced at a density of 10,000 cells/cm^2^ have a higher proliferation rate between day 1 and day 2 (3.32 vs. 2.07; *p*-value < 0.001) and a lower proliferation rate between day 2 and day 3 (2.01 vs. 3.73; *p*-value < 0.001) (Fig. 4a). In contrast, cells grown at low density continue to increase their proliferation rate over the 3 days of induction. Notably, while VPCs are not contact-inhibited, cell cultures at all seeding densities remain subconfluent after 3 days of culture (Fig. 1D) and continue to proliferate. A3-ESCs seeded at the highest density contained fewer cells of a small diameter representative of ESC size three days post induction compared with cells seeded at lower density (5–6 μm, 26% vs 36%; *p*-value < 0.001). Additionally, proportionately more cells of larger diameter were found in cultures seeded at a density of 10,000 cells/cm^2^ compared with lower density (9–10 μm, 20% vs 8%; *p*-value < 0.001) (Fig. 4b). The forward scatter measurements from fluorescence-activated cell sorting in flow cytometry, another indication of cell size, show that the early A3-ESCs are smaller compared with the larger differentiated FLK1^+^ VPCs (Fig. 4c).

### Differential gene expression of cell-to-cell signaling molecules during density-dependent differentiation

**Fig. 5:**
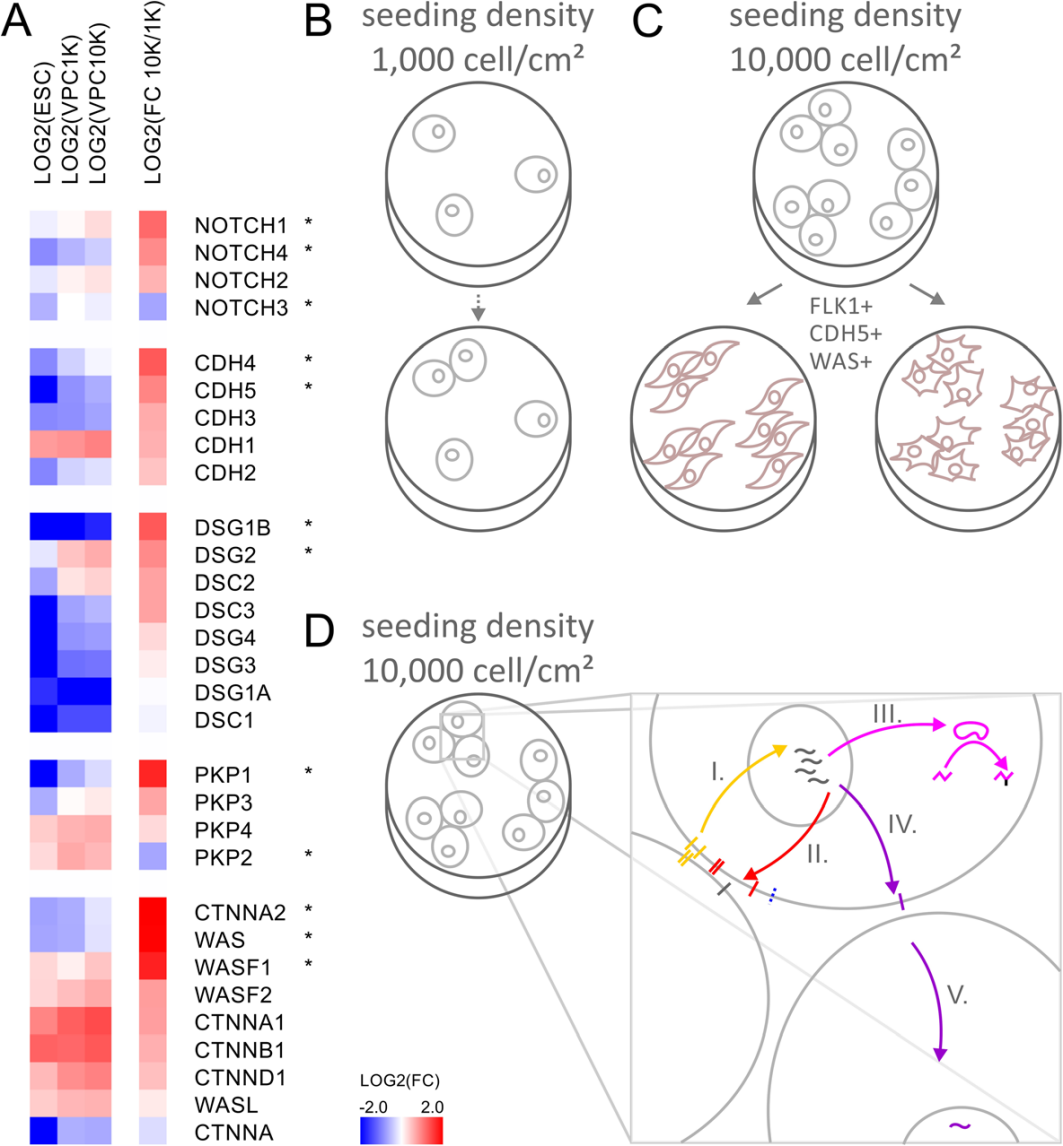
Differential gene expression analysis of density-dependent differentiation identifies molecules related to cellular adhesion and vascular genesis. **a** Gene expression analysis of embryonic stem cells (ESCs) as well as vascular progenitor cells (VPCs) seeded at density of 1,000 vs 10,000 cells/cm^2^. The logarithmic fold change of cells seeded at 10,000 cells/cm^2^ in comparison to 1,000 cells/cm^2^ (LOG2(FC 10 K/1 K)) identifies density-depended processes. Gene symbols labeled with an asterisk * indicate significant differential expression with *p*-values below 0.05. Schematic model compares: **b** Poor differentiation efficiency of embryonic stem cells seeded at low density of 1,000 cells/cm^2^, where isolated cells eventually die off. **c** Seeding of embryonic stem cells at high density of 10,000 cells/cm^2^ results in expression of differentiation markers of vascular progenitor cells, which are a potent starting point for tissue engineering. **d** Enlarged diagram of cell-cell contact shows signaling processes and positive feedback enhancing differentiation: I. Cell-cell contacts mediate signals into nucleus. II. Transcriptional changes create positive feedback enforcing cell surface contacts and stimulating regulators of adherens junctions and desmosomes. III. Switch of metabolism from exponential, proliferative mode to differentiated phenotype. IV. Enforcement of new cell surface contacts. V. Propagation of differentiation state to neighboring cells

In order to further investigate the density-dependent signaling directing FLK1^+^ VPCs, a number of cell-to-cell signaling molecules were examined using a targeted PCR array. The expression pattern of cells seeded at densities of 10,000 cells/cm^2^ or 1,000 cell/cm^2^ revealed significant differential expression with *p*-values below 0.05 and a fold change of 2.0 or higher (Fig. 5a). Gene expression pattern included significant up-regulation of NOTCH1 (GeneBank: 4851), NOTCH4 (GeneBank: 4855), CDH4, cadherin 4, retinal, R-cadherin (GeneBank: 1002), CDH5, cadherin 5, vascular endothelium, VE-cadherin (GeneBank: 1003), DSG1B, desmoglein 1 (GeneBank: 1828), DSG2, desmoglein 2 (GeneBank: 1829), PKP1, plakophilin 1 (GeneBank: 5317), CTNNA2, catenin cadherin-associated protein alpha 2 (GeneBank: 1496), WAS, Wiskott-Aldrich syndrome (GeneBank: 7454), and WASF1, WAS protein family, member 1 (GeneBank: 8936) as well as significant down-regulation of NOTCH3 (GeneBank: 4854), and PKP2, plakophilin 2 (GeneBank: 5318).

### Differential protein expression of cell-to-cell signaling molecules during density-dependent differentiation

**Fig. 6:**
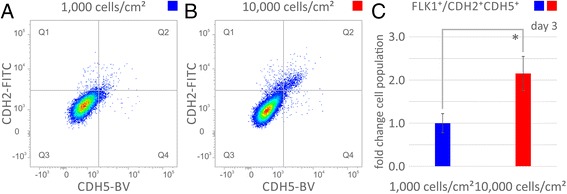
Differential protein expression of cell adhesion molecules during density-dependent differentiation identifies molecules related to cellular adhesion and formation of vascularization. Protein expression analysis of induced vascular progenitor cells (VPCs) derived from embryonic stem cells (ESCs) comparing seeding densities of 1,000 vs 10,000 cells/cm^2^. Flow cytometric cell scanning plots of 100,000 events of differentiating FLK1^+^ embryonic stem cells (**a**) seeded at low density of 1,000 cells/cm^2^ and (**b**) seeded at high density of 10,000 cells/cm^2^. The FLK1^+^ cells were gated in quadrant 1 (Q1) for FLK1^+^/CDH2^+^CDH5^−^, in quadrant 2 (Q2) for FLK1^+^/CDH2^+^CDH5^+^, in quadrant 3 (Q3) for FLK1^+^/CDH2^−^CDH5^−^, and in quadrant 4 (Q4) for FLK1^+^/CDH2^−^CDH5^+^. Markers were quantified by fluorescence of FLK1 allophycocyanin conjugate (APC), CDH2 fluorescein isothiocyanate (FITC) conjugate and CDH5 brilliant violet (BV) conjugate. **c** Fold change of FLK1^+^/CDH2^+^CDH5^+^ gated in Q2 with significant differential expression of cells derived from seeding at densities of 1,000 vs 10,000 cells/cm^2^ indicated by asterisk * with *p*-value below 0.05

Protein level differences in cell-to-cell signaling molecule expression were quantified by flow cytometry (Fig. 6). Induced VPCs originally seeded at 1,000 cell/cm^2^ or 10,000 cells/cm^2^ were stained and analyzed for FLK1, CDH2 (cadherin 2, neuronal, N-cadherin, GeneBank: 1000) and CDH5 expression. Importantly, the cell adhesion molecule CDH5, VE-cadherin, is indicative of vascular endothelial differentiation. The percentage of cells staining positive for FLK1, CDH2, and CDH5, FLK^+^/CDH2^+^CDH5^+^, quadrant 2, (Fig. 6a-b) was higher for cells originally seeded at 10,000 cells/cm^2^ than those seeded at 1,000 cells/cm2 (1.51% vs 0.70%, *p* < 0.01) (Fig. 6c).

## Discussion

The generation of FLK1^+^ VPCs from ESCs peaks on day 3, followed by a reduction in FLK1^+^ numbers (Fig. 2ab) within the range of reported days (2–5) during mesoderm induction from ESCs [15, 17, 22–25]. The other significant variable in the efficient induction of VPCs was a high cell seeding density (Fig. 2a-b), while VEGF treatment level was not significant. The higher density and robustly differentiating VPC cultures also correlated with reduced proliferation rates and greater cell diameters, both indicative of differentiation (Fig. 4). Although these cells are not contact inhibited nor were they confluent cultures, hypoxia is known to drive mesoderm commitment [26, 27] and endothelial fate [19] from ESCs. To determine whether hypoxia could drive ESC differentiation, we calculated the molar fraction of oxygen at the cell surface of our high density cell dishes cultures. Using our experimental cell proliferation rates, estimated oxygen solubility in saline solution, and oxygen consumption rates reported for both ESCs = 27.5x10^−18^ [28] and ECs = 50x10^−18^ mol/cell/s [20], it was determined that, although oxygen would be reduced at higher cell seeding densities, none of the conditions would generate a hypoxic environment (defined as 1-3% oxygen).

Interestingly, VEGF treatment was not a determining or statistically significant variable in directing VPC fate. A large body of data implicates VEGF signaling in mesoderm and endothelial cell fate and that the FLK1/VEGF receptor is one of the key markers defining the angioblast cell [12, 14, 29, 30]. However, since BMP4 signaling can also activate the VEGF/VEGFR signaling [31, 32], it is sufficient in the inductions shown. Moreover, the two distinct VEGF binding domains in the fibronectin matrix [33] may stabilize and protect autocrine VEGF production from degradation [33], as well as, aid in cell presentation. The presumptive requirement of VEGF treatment in chemically-defined media for EC fate has been most rigorously examined using single cells cultured in collagen-type IV coated 96-well plates [12, 30, 34]. Without fibronectin matrix to sequester and protect the VEGF generated by the cells, one might expect that VEGF treatment would be required in these cultures. However, our results suggest that the utilization of fibronectin matrix mitigates the need for VEGF supplementation in VPC induction cultures.

Differential gene expression array analysis identified a number of cell-to-cell signaling molecules that were up-regulated in the higher density cultures containing more VPCs. Differential expression of cell surface receptors, desmosome, catenins, and cytoskeleton regulators could be required for, or facilitate, the density-dependent differentiation of ESCs (Fig. 5b-d). Vascular cells take advantage of many different cell adhesion contacts demonstrated by the global up-regulation of cadherins, desmosomal and desmoglein components. Since cell surface molecules have the ability to communicate extracellular changes into the cytosol, such as contact formation with neighboring cells, the gene expression data suggests a positive feedback reinforcing cellular contacts (Fig. 5d). Initial cell-cell contacts mediate signals into the nucleus, where transcriptional changes create positive feedback promoting and strengthening cell surface contacts and stimulating regulators of adherens junctions and desmosomes. Positive feedback circuits have the ability to create threshold densities for successful differentiation. Once established and supported by the cell type specific cell surface contacts molecules, signals of differentiation can lead to lineage committed cell fates and organized tissue formation.

Amongst genes exhibiting the strongest change in the context of seeding density-dependent differentiation of VPCs were the cytoskeleton regulators WAS and CTNNA2. The expression pattern for both genes is unaffected in the lower density cohort but was consistently up-regulated in the higher density cohort. Wiskott-Aldrich syndrome protein (WASP) is a key regulator of endothelial cell-cell junctions and cytoskeleton dynamics and helps form and maintain the integrity and function of EC monolayers [35]. Moreover, WASP organizes actin and vascular epithelium-cadherins at EC junctions, and hence is vital for the assembly of vascular structures [35–37]. Importantly, along with FLK1 expression, WAS and CDH5 are also indicators of vascular differentiation [8]. Other studies have identified members of the E-twenty six (ETS) transcription factor family associating with FLK1 and CDH5 promoters in vascular epithelia to regulate vascular specification from primitive mesoderm [8–10, 38]. In murine and amphibian model organisms, plakophilins have been found localized to the nucleus of ESCs and form complexes with members of the ETS family of transcription factors to direct development related gene transcription events [39].

Similar to PKP1, CTNNA1, catenin cadherin-associated protein, alpha 1 (GeneBank: 1495) and AJAP1, adherens junctions associated protein 1 (GeneBank: 55966) expression levels are correlated with advancing tumor stage and inversely related to cell proliferation [32, 40, 41]. While CTNNA2 has been found as hub for extracellular matrix organization, loss of CTNNA1 is exhibited by multiple cancer types, and restoration of CTNNA1 expression in acute myeloid leukemia cells led to lower proliferation [27, 40]. Additionally CTNNA1 regulates differentiation events in the developing nervous system by maintaining beta-catenin signaling [42]. It is possible that the higher levels of PKP1 and CTNNA1 seen in the 10,000 cells/cm^2^ density group causes these cells to slow their proliferation in favor of differentiation and growth. The regulation of desmosomal assembly by DSG1B, DSG2, and PKP1 not only enforces cell surface adhesion contacts between ECs but also regulates the cell signaling events in the cytoplasm and nucleus. DSG2 regulates actin assembly in ECs and affects proliferation via modulation of EGFR signaling [43, 44]. PKP1 associates with eukaryotic translation initiation factor 4A1 to stimulate protein translation [45] and loss of PKP1 is linked to prostate cancer proliferation [46]. Nuclear PKP1 complexes with catenin and is found bound to single stranded DNA [47]. PKP2, which is more abundant in the lower density group, binds to catenin and complexes with the RNA polymerase III holoenzyme [48].

The cadherin family uniformly responds to density-dependent differentiation [36, 37]. All cadherins assayed show up-regulation in the higher density cohort. CDH4 shows the highest density-dependent fold-change of the cadherin family. In addition to significant density-dependent up-regulation, vascular endothelial CDH5 is also significantly different between undifferentiated and induced ESCs. Of the desmosomal, desmocollin, and desmoglein components, DSG1B, DSG2, and PKP1 stand out as positive responders to density-dependent differentiation supporting formation of cell surface adhesion contacts in endothelial formation. For the majority of cell surface, cell junction and desmosomal components, a global increase in gene expression in response to density-dependent seeding is observed.

Among the differentiation and density-dependent effects on gene expression, perhaps the most profound is differential expression of the NOTCH receptor family. NOTCH signaling is a highly conserved intercellular signaling mechanism essential for proper cell fate choices during development [49]. Both NOTCH1 and NOTCH4 have both been implicated in vascular morphogenesis [50]. Moreover, NOTCH1 is found expressed in both endothelial and hematopoietic progenitor cells [51], while NOTCH4 is expressed in ECs, but not in hematopoietic progenitor cells [50]. NOTCH3 signaling is highest in late stage smooth muscle cell differentiation [52] and neural differentiation [53]. In the high cell density cultures, NOTCH1 and NOTCH4 were significantly up-regulated, while NOTCH3 is significantly down-regulated at induction conditions of 10,000 cells/cm^2^ compared with the lower density cultures.

It is expected that differential expression of NOTCH components within ESCs seeded at higher density is, at least indirectly, responsible for the shift in metabolite utilization observed during the differentiation process. Specifically, NOTCH signaling has recently been linked to the regulation of cellular metabolism [54, 55], inducing glutamate uptake during the terminal differentiation of astrocytes [54]. Furthermore, NOTCH inhibition in glioma stem cells led to reductions in intracellular glutamate and glutamine, and increased lactate and threonine [55]. In the same study, it was noted that NOTCH blockade modulated the expression of multiple genes regulating glutamate metabolism, including glutaminase and several glutamate transporters [55]. Tight regulation of glutaminase activity and glutamate metabolism are vital features of both stem cell function and tumor survival [11, 30, 44]. Interestingly, glutamine metabolism also regulates chromatin structure and pluripotency related transcription factors, such as OCT4, and therefore may play a pivotal role in vascular development [20]. Additional studies examining the role of cell-cell signaling components, particularly NOTCH, in the regulation of glutaminergic and other metabolic pathways could help optimize strategies for ESC differentiation and understand NOTCH-mediated cancer progression.

An increase in cell size correlating with stem cell differentiation is intimately coupled to loss of “stemness”. Moreover, larger cells proliferate more slowly compared to smaller cells [56]. While in cancer cells, a positive feedback is used to rapidly ramp up a distinct metabolic program [57], cellular differentiation is accompanied by a switch in metabolism from an exponential proliferative mode into a differentiated phenotype. During the time course of differentiation, ESCs start out as small, rapidly dividing cells, but rapidly shift away from exhaustive glycolysis and high metabolite consumption to a reduced metabolic demand per cell. This observed switch in metabolism also supports the changing demands of larger, more differentiated VPCs. Our data shows that the gene expression program of these differentiating ESCs also dynamically responds to the culture conditions at higher cellular density, and actively reinforces cell surface signaling components leading to up-regulation of genes associated with VPC fate. This strengthening of cellular communication may help regulate the concurrent switch of metabolism from an exponential, proliferative mode to a differentiated, growth permissive phenotype.

## Conclusions

In summary, we have identified a density-dependent metabolic shift correlating with increased differentiation of VPCs from ESCs. This density-dependent differentiation model is associated with reduced cellular metabolism, highlighted by a decrease in exhaustive glycolysis, by a decrease in proliferation, and by an increase in cell size. Concomitant is enhanced expression of cell-cell signaling components, including those known to regulate the differentiation and metabolism of stem cells via density-sensing positive feedback circuits. In the future, efficient tissue engineering approaches may take advantage of such density-dependent switches and control crosstalk between cell-cell signaling and cellular metabolism.

## Abbreviations

CSCs: Cancer stem cells
CT: Threshold cycle
ECs: Endothelial cells
ESCs: Embryonic stem cells
ETS: E-twenty six
FBS: Fetal bovine serum
GOI: Gene of interest
HKG: Housekeeping genes
iPSCs: Induced pluripotent stem cells
KO-DMEM: Knockout Dulbecco’s Modified Eagle Medium
KSR: Knockout serum replacer
LIF: Leukemia inhibitory factor
MEFs: Mouse embryonic fibroblasts
MEM: Minimal essential medium
PBS: Phosphate buffered saline
QPCR: Quantitative polymerase chain reaction
RT: Real-time
TSP: (trimethylsilyl)propanoic-2,2,3,3-d4 acid
VPCs: Vascular progenitor cells
WASP: Wiskott-Aldrich syndrome protein
ΔCT: Difference threshold cycle value

## Used gene symbols

ACTB: actin beta, GeneBank: 60
AJAP1: adherens junctions associated protein 1, GeneBank: 55966
B2M: beta-2-microglobulin, GeneBank: 567
BMP4: bone morphogenetic protein 4, GeneBank: 652
CDH2: cadherin 2, type 1, neuronal, N-cadherin, GeneBank: 1000
CDH4: cadherin 4, type 1, retinal, R-cadherin, GeneBank: 1002
CDH5: cadherin 5, type 2, vascular endothelium, VE-cadherin, GeneBank: 1003
CTNNA1: catenin (cadherin-associated protein), alpha 1, GeneBank: 1495
CTNNA2: catenin (cadherin-associated protein), alpha 2, GeneBank: 1496
DSG1B = DSG1: desmoglein 1, GeneBank: 1828
DSG2: desmoglein 2, GeneBank: 1829
FLK1 = VEGFR = KDR: kinase insert domain receptor, GeneBank: 3791
GAPDH: glyceraldehyde-3-phosphate dehydrogenase, GeneBank: 2597
GUSB: glucuronidase beta, GeneBank: 2990
HSP90AB1: heat shock protein 90kDa alpha class B member 1, GeneBank: 3326
NOTCH1: notch1, GeneBank: 4851
NOTCH3: notch3, GeneBank: 4854
NOTCH4: notch4, GeneBank: 4855
PKP1: plakophilin 1, GeneBank: 5317
PKP2: plakophilin 2, GeneBank: 5318
POU5F1: OCT3/4, POU class 5 homeobox 1, GeneBank: 5460
VEGFA = VEGF: vascular endothelial growth factor A, GeneBank: 7422
WAS: Wiskott-Aldrich syndrome, GeneBank: 7454
WASF1: WAS protein family, member 1, GeneBank: 8936.
